# The First Documented Ibuprofen-Induced Toxic Epidermal Necrolysis in the Middle East and North Africa Region: A Case Report, Complications, and Management

**DOI:** 10.7759/cureus.49608

**Published:** 2023-11-28

**Authors:** Karim Kheir, Rim M Abdallah, Ziad Sleiman, Hassan Mallat, Fady Haddad

**Affiliations:** 1 Department of General Medicine, Faculty of Medical Sciences, Lebanese University, Beirut, LBN; 2 Department of Allergy and Immunology/Internal Medicine, Faculty of Medical Sciences, Lebanese University, Beirut, LBN; 3 Department of Plastic and Reconstructive Surgery, Lebanese Hospital Geitaoui - University Medical Center, Beirut, LBN; 4 Department of Infectious Diseases, Doctoral School of Sciences and Technology, Faculty of Public Health, Lebanese University, Tripoli, LBN; 5 Department of Internal Medicine and Clinical Immunology, Lebanese Hospital Geitaoui - University Medical Center, Beirut, LBN

**Keywords:** ibuprofen, severe cutaneous adverse reactions, immunology, dermatology, toxic epidermal necrolysis

## Abstract

Introduction: Stevens-Johnson syndrome (SJS), Stevens-Johnson/toxic epidermal necrolysis overlap syndrome (SJS/TEN) and toxic epidermal necrolysis (TEN) are rare, acute, potentially lethal conditions, considered to be part of the severe cutaneous adverse reactions (SCARs) spectrum, with TEN being the most life-threatening. The distinction between these three entities is based on the extent of total skin surface involvement, with SJS involving < 10%, SJS/TEN involving 10-30% and TEN involving > 30% of total body surface area. These mucocutaneous reactions are most commonly caused by a hypersensitivity reaction to a drug, with infections and vaccines being possible, less common etiologies.

Case presentation: In the following case report, we summarize a rare case of a 43-year-old, previously healthy male patient who presented with TEN after taking ibuprofen, a non-steroidal anti-inflammatory drug. According to PubMed literature, this is the first documented case of ibuprofen-induced TEN in the Middle East and North Africa (MENA) region.

Discussion: TEN is an autoimmune bullous disorder that results in the death of keratinocytes, leading to complete dermo-epidermal separation. In the case of our patient, the desquamation was extensive, involving 70% of the total body surface area, and was complicated by a triple bacterial infection with *Acinetobacter baumannii*, *Klebsiella pneumoniae*, and *Pseudomonas aeruginosa*. The patient was treated with colistin and meropenem, in addition to supportive management, hydration and nutritional support.

Conclusion: In the case of TEN, early diagnosis and hospitalization in a burn centre are crucial to allow rapid healing, and improve the quality of life of the affected patients. Immediate cessation of the causative mediation is critical. Supportive management, hydration, nutritional support, and maintenance of aseptic conditions are highly encouraged to reduce the mortality and morbidity associated with TEN.

## Introduction

Toxic epidermal necrolysis (TEN) is a rare, severe mucocutaneous reaction, estimated to affect 0.4-1.9 cases per million worldwide, each year [1]. In the majority of cases, TEN results from a type IV hypersensitivity reaction to a drug, although infection and vaccination are potential causes [2].

Affected patients usually present with fever and a non-specific, flu-like prodrome, followed by the appearance of pruritic, targetoid lesions, covering > 30% of total body surface area [3]. These cutaneous findings rapidly evolve to form painful, erythematous blisters, causing desquamation of the skin and mucous membranes, such as the oral, genital and ocular surfaces. Blisters, erosions, sloughing and oozing are common mucocutaneous findings in TEN. A higher occurrence of severe cutaneous adverse reactions has been reported among patients with HIV-1 infection [3], systemic lupus erythematosus [3], and *Mycoplasma pneumoniae* [4]. In severe cases, involvement of the internal organs, including the lungs and the intestines, is seen and is associated with a poor prognosis [5].

## Case presentation

In the following case report, we present the case of a 43-year-old, previously healthy male patient, who developed TEN after taking ibuprofen. The patient does not take any chronic medications and has no previously known drug allergies. He reported falling on his back, with no documented spine fractures. While reviewing his drug history, the patient mentioned taking a total of seven oral tablets of ibuprofen 400 mg recently, to manage his back pain, before the onset of the rash associated with TEN.

**Figure 1:**
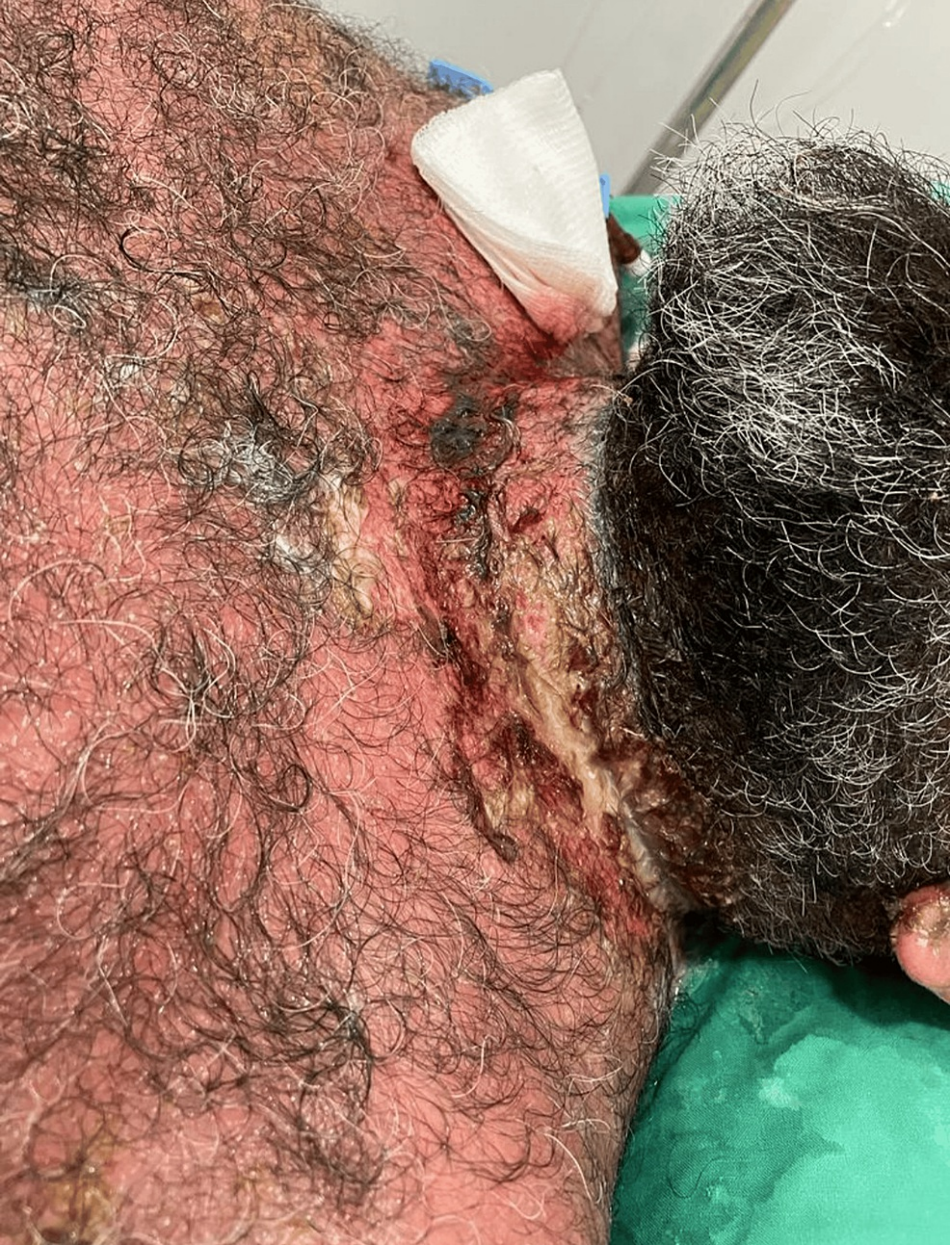
The beginning of the rash on the patient's neck

After taking ibuprofen, the patient reported a fast-spreading rash that started on his neck (Figure 1), and then quickly covered different areas of his body, for which he took desloratadine/betamethasone pills. The rash consisted of erythematous macules that quickly spread and coalesced, and eventually blistered, resulting in oozing and painful, raw areas of skin. When the rash kept spreading further, the patient was given two intramuscular injections of triamcinolone 40 mg. Despite all these measures to control the severe cutaneous reaction, the patient’s rash kept exacerbating, and hospitalization was recommended for further follow-up.

The patient was first admitted to the ICU in another hospital where a central venous catheter was placed. He was started on piperacillin/tazobactam and steroids for a period of five days. He was then transferred to Geitaoui Hospital - University Medical Center (UMC). On admission to the latter, the vitals were measured: the patient’s temperature was 38.3°C (100.9°F), with a heart rate of 104 bpm, and a respiratory rate of 23 breaths/min. Moreover, his blood pressure was 137/69 mmHg, with an oxygen saturation of 91%.

Initial blood tests showed the following results (Table 1):

**Table 1:** The initial blood test results at the Geitaoui Hospital - University Medical Center BUN: blood urea nitrogen

	Results	Minimum	Maximum
White blood cell count (cells/μL)	4960	4800	10800
Hemoglobin (g/dL)	9.2	14	18
Neutrophil percentage (%)	71	60	70
Lymphocyte percentage (%)	27.8	23	30
Platelets count (platelets/μL)	292000	130000	400000
Sodium (mmol/L)	140	136	145
Potassium (mEq/L)	4.11	3.5	5.1
BUN (mg/dL)	15	8	25
Creatinine (mg/dL)	0.62	0.61	1.24

In addition to these parameters, the serum bicarbonate level on admission to our hospital was 30.5 mmol/L, with the normal range being 22-27 mmol/L. However, this value represents the corrected bicarbonate level, since it was already corrected in the previous hospital. As the patient was admitted to our hospital seven days after the acute onset of TEN, and since the uncorrected bicarbonate level in the acute phase is an essential criterion of the SCORTEN score to predict the mortality rate [6], this score could not be calculated in the case of our patient.

**Figure 2:**
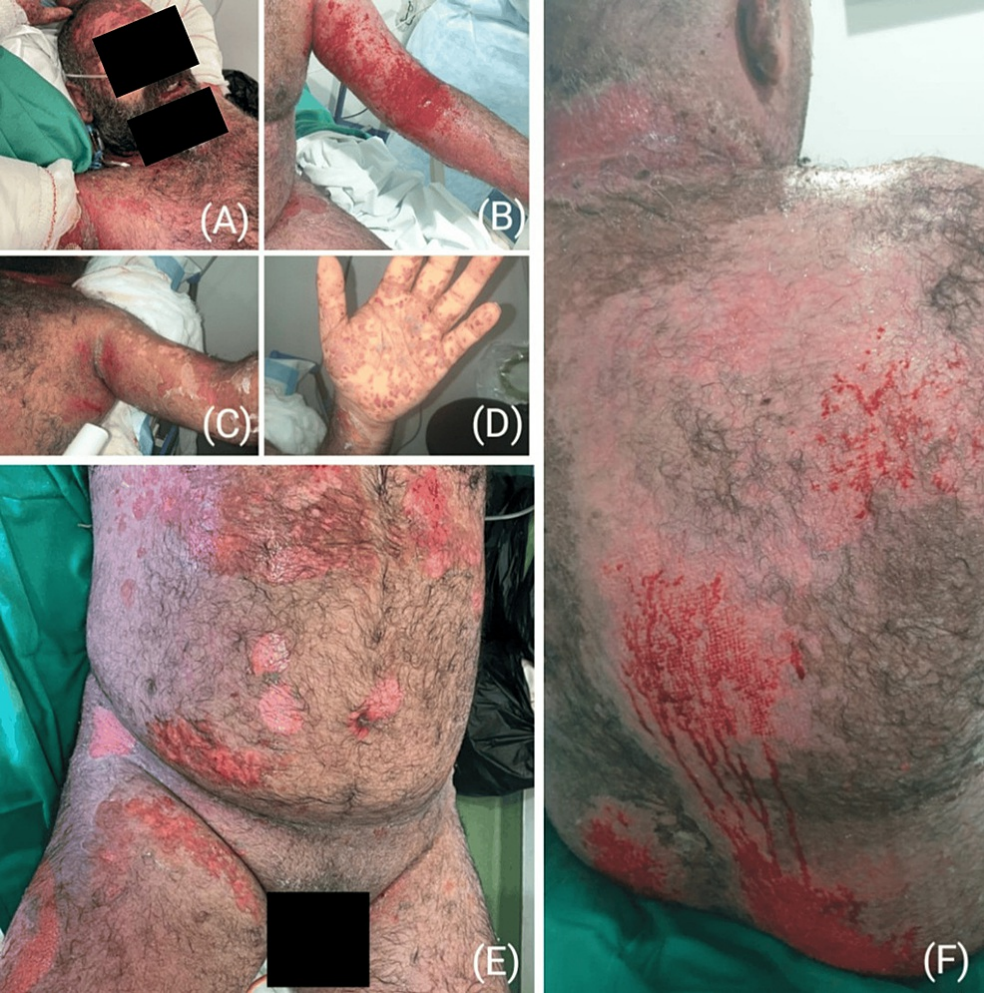
The multiple cutaneous lesions on the face, lips, upper limbs, chest, abdomen, back and thighs of the patient, on arrival to the Burn Center at the Geitaoui Hospital - University Medical Center

On inspection, the affected total body surface area was around 70%. The rash was seen on the face, neck, chest, abdomen, back, buttocks, hands and legs, as well as on the oral, corneal and penile mucosa (Figure 2). Extensive, full-thickness exfoliation of the epidermis and mucous membranes was obvious, and Nikolsky’s sign was clearly positive. The patient was transferred to the burn centre where a short course of methylprednisolone IV was started at a dose of 40 mg every eight hours. He was also given clindamycin IV 600 mg every six hours, as well as ipratropium bromide to manage his breathing difficulties.

In the burn centre, the patient was febrile and in intense pain. He was isolated in fully aseptic conditions. Dressings were performed every 48 hours, using petroleum jelly and paraffin gauze. Morphine was given to reduce the patient’s severe pain prior to each dressing session. The mucocutaneous lesions showed significant improvement after each dressing session, and both erythema and blistering became progressively less noticeable over the course of three to four weeks.

**Figure 3:**
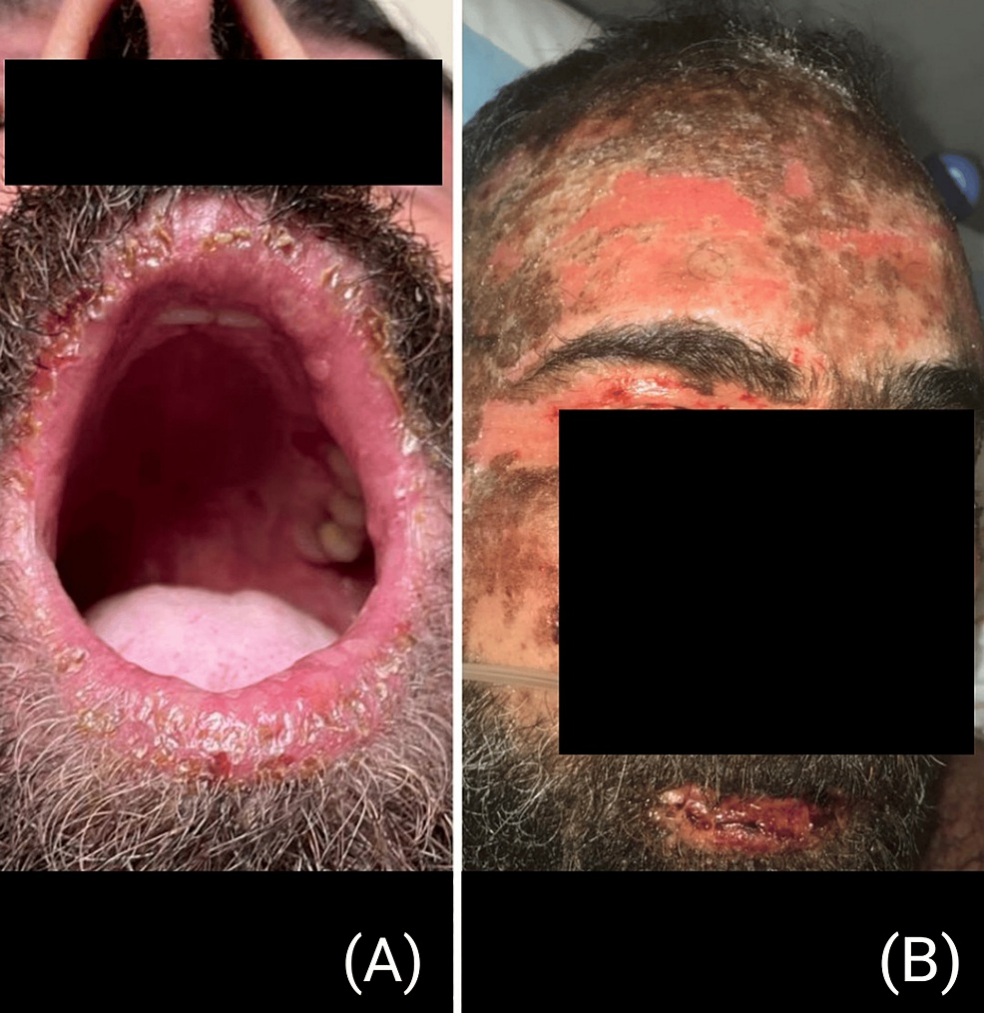
The progression of the exfoliative crusts on the patient's lips

Initially, parenteral nutrition, as well as IV fluid resuscitation, were started, since the patient was suffering from relentless dysphagia and odynophagia. On physical examination, the patient had multiple, exfoliative lesions on the buccal mucosa and extensive, blackish hemorrhagic crusts on the lips (Figure 3). The severe peeling and denudation of the lips and the perioral region were complicated by an oral fungal infection. Anidulafungin was given intravenously for a total of 10 days, with routine mouthwash care, and significant improvement and re-epithelialization of the lips were seen after a couple of weeks. Soft foods and fluids were only given once the oral lesions subsided, and solid foods were resumed 16 days after admission to the burn centre when the oral lesions were fully healed.

Additionally, erythema and dryness were noted on the ocular mucosa of both eyes. The patient was put on artificial tear drops for ocular lubrication since the first day of admission, following a consultation with an ophthalmologist. Fortunately, the redness faded away within a few days, and no additional ocular complications were reported.

On day 2 following admission to the Geitaoui Hospital - UMC, both blood and wound cultures revealed the presence of extensively drug-resistant *Acinetobacter baumannii*, with a procalcitonin level exceeding 100 ng/ml, indicating a high risk of mortality [7], while urine culture only showed a positive result for the same bacterium on day 4. At this stage, the patient was started on colistin IV 4.5 million international units every 12h and meropenem IV 1g every 8h. Moreover, on day 7 post-admission, both blood and central venous catheter tip cultures turned out to be positive for *A. baumannii* and *Klebsiella pneumoniae*.

Finally, a wound culture was performed on day 9 and revealed the presence of *Pseudomonas aeruginosa*. After waiting for another two days, cultures were performed again and came back positive for *A. baumannii* only. Colistin and meropenem were continued until the patient was discharged home.

**Figure 4:**
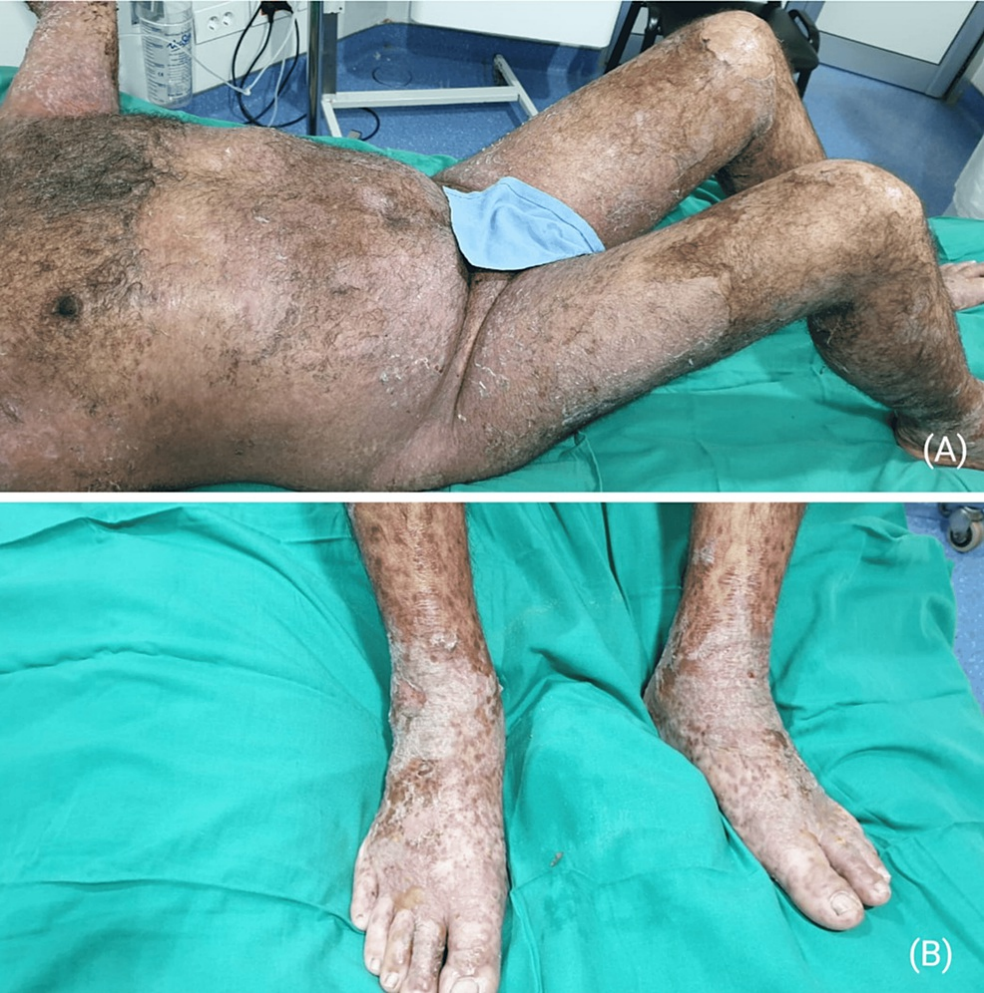
The residual pigmentary changes on the patient's abdomen and lower limbs before discharge from the hospital.

After 28 days of hospitalization at the burn centre in the Geitaoui Hospital - UMC, the patient was successfully discharged home. His cutaneous lesions recovered, but residual pigmentary changes and scarring remained, especially on his lower limbs (Figure 4).

## Discussion

Although the precise pathogenesis of TEN is poorly established, TEN is believed to be an acute, type IV hypersensitivity reaction, most commonly induced by medications such as allopurinol, sulfonamides, carbamazepine, phenytoin, and nonsteroidal anti-inflammatory drugs (NSAIDs) [8]. These causative drugs can bind to both major histocompatibility complex class I (MHC-I) and T-cell receptors, leading to a clonal expansion of cytotoxic T cells, which accumulate in the epidermal blisters and attack the keratinocytes, resulting in apoptosis [9]. Hence, TEN is considered to be provoked by an immune, allergic reaction to an antigenic drug-host tissue complex [10], and is predominantly triggered by CD8+ T lymphocytes, in addition to natural killer cells [9]. Moreover, soluble death mediators and cytotoxic molecules, especially granulysin, have been associated with the blistering and the death of keratinocytes seen in patients with TEN [11].

Regarding the generation of the antigenic drug-host tissue complex, three theories have been suggested to explain the pathogenesis of TEN: (1) covalent binding of the culprit medication to a cell-surface peptide, known as the hapten/pro-hapten model; (2) non-covalent binding of the causative medication to a specific MHC-I, known as the p-i theory; and (3) direct binding of the medication to MHC-I, with formation of an aberrant drug-modified human leukocyte antigen (HLA)-peptide repertoire that enhances the likelihood of autoimmune reactions, known as the altered peptide theory [10]. Nevertheless, since particular HLA alleles have been associated with an increased risk of developing TEN, the second and third models, which are HLA-restricted theories, are more consistent with the concept of genetic predisposition to drug-induced TEN [10].

In many cases, the HLA system is particularly involved in the pathophysiology of TEN. For instance, patients with HLA-B*5801 have an increased risk of developing allopurinol-induced Stevens-Johnson syndrome (SJS)/TEN, especially among the Thai and Han Chinese populations [12], while those with HLA-B*1502 are at a higher risk of developing carbamazepine-induced SJS/TEN, particularly in Han Chinese, Thai, and Malaysian ethnicities [13].

When it comes to the Middle East and North Africa (MENA) region, few studies related to TEN exist in the literature, due to the rarity of this condition. In a study conducted on 12 patients diagnosed with TEN in the United Arab Emirates, it was found that the most culprit drugs causative of TEN were beta-lactam antibiotics, accounting for 75% of the reported TEN cases, while one case of TEN was reported with each of the following drugs: cefuroxime, diclofenac, lansoprazole, and rofecoxib [14]. A similar study conducted in Saudi Arabia involved 10 patients diagnosed with SJS/TEN, and found that antibiotics were associated with five out of the ten reported cases: three cases were induced by amoxicillin/clavulanic acid, one case was caused by azithromycin, and another case was due to ciprofloxacin. Among the remaining cases, two were due to carbamazepine, one case was attributed to levetiracetam, another was due to acetaminophen, with one remaining case being provoked by an unknown over-the-counter drug [15].

While NSAIDs and allopurinol were considered to be the most common drugs causative of TEN in France and Austria respectively, studies conducted in Saudi Arabia and the United Arab Emirates found antibiotics, particularly beta-lactams, to be the most common medications causative of TEN in the MENA region, followed by antiepileptic drugs [14,15]. However, no previous cases of ibuprofen-induced TEN in the MENA region have been found on PubMed, making this case report the first of its kind.

In the case of our patient, the involvement of 70% of the total body surface area was indicative of a life-threatening condition. As the diagnosis of TEN is essentially clinical [16], our diagnosis was based on the clinical findings seen in our patient: the rapidly progressive rash extending to the mucous membranes, with the typical blisters, epidermal detachment and targetoid lesions seen in TEN, triggered by the intake of a new medication. The positive Nikolsky sign was also helpful in eliminating other possible diagnoses. As the blisters covered more than 30% of the total body surface area, the diagnosis was in favour of TEN, rather than other blistering entities, such as SJS or SJS/TEN overlap syndrome [3].

Since initially, the oral mucosa was significantly involved, parenteral nutrition was preferred. The latter was then replaced by enteral feeding once oral re-epithelialization was achieved, in order to reduce the risk of bacterial translocation associated with parenteral feeding.

In addition, due to the high risk of nosocomial infections in patients with TEN, continuous screening for possible infections was conducted in the case of our patient, in order to treat any bacterial or fungal infections without further delay. After admission to the burn centre, cultures were routinely taken every other day from multiple sites, and bacterial and fungal infections were successfully managed with colistin, meropenem, and anidulafungin. The most life-threatening bacterial infection detected in this case was caused by *A. baumannii*, which turned out to be an extensively drug-resistant strain. In fact, *A. baumannii* is considered to be one of the most challenging gram-negative bacteria to manage and treat and is more likely to affect patients with prolonged lengths of hospitalization, as well as those receiving wound care procedures [17]. According to the literature, it has been shown that combining colistin with a carbapenem was associated with higher rates of success than any other antibiotic used with colistin, when dealing with extensively-drug resistant *A. baumannii* infections [18], confirming the appropriate management in the case of our patient, who was treated with colistin and meropenem. With suitable handling, patients with *A. baumannii* infections receiving colistin can have rates of cure ranging from 57% to 77% [17].

Finally, given that the cases of TEN are relatively scarce in the literature, no definitive therapeutic modalities have been shown to be of proven efficacy, and the use of corticosteroids and intravenous immunoglobulin, among other therapeutic approaches, is still grossly based on observational studies [19]. However, we strongly believe, as supported by the literature [20], that early hospitalization, with supportive management, hydration, and nutritional support had a major role in reducing the mortality and morbidity in our patient.

## Conclusions

In conclusion, we described the first documented case of toxic epidermal necrolysis induced by ibuprofen in the MENA region. This rare condition must be diagnosed early for rapid follow-up of affected patients. In the case of a drug-induced toxic epidermal necrolysis, the causative medication must be immediately discontinued. Admission to a burn centre without further delay is highly recommended for proper management and reduction of the complications and mortality associated with TEN. A multi-disciplinary approach of affected patients is preferred, to allow rapid and optimal healing, especially when multi-organ involvement is suspected. Since affected patients are vulnerable to infections, asepsis must be maintained continuously to reduce the risk of nosocomial infections. Supportive management, hydration, and nutritional support, as well as pain management, are crucial to improve the outcomes and the quality of life.
